# Using a Qualitative Phenomenological Approach to Inform the Etiology and Prevention of Occupational Heat-Related Injuries in Australia

**DOI:** 10.3390/ijerph17030846

**Published:** 2020-01-29

**Authors:** Alana L Hansen, Susan Williams, Scott Hanson-Easey, Blesson M Varghese, Peng Bi, Jane Heyworth, Monika Nitschke, Shelley Rowett, Malcolm R Sim, Dino L Pisaniello

**Affiliations:** 1 School of Public Health, The University of Adelaide, Adelaide, SA 5005, Australia; alana.hansen@adelaide.edu.au (A.L.H.); susan.williams@adelaide.edu.au (S.W.); scott.hanson-easey@adelaide.edu.au (S.H.-E.); Blesson.varghese@adelaide.edu.au (B.M.V.); peng.bi@adelaide.edu.au (P.B.); 2 School of Population and Public Health, The University of Western Australia, Crawley, WA 6009, Australia; jane.heyworth@uwa.edu.au; 3 Department for Health and Wellbeing, Government of South Australia, 11 Hindmarsh Square, Adelaide, SA 5000, Australia; Monika.Nitschke@sa.gov.au; 4 SafeWork SA, Government of South Australia, 33 Richmond Road, Keswick, SA 5035, Australia; Shelley.Rowett@sa.gov.au; 5 Department of Epidemiology and Preventive Medicine, School of Public Health and Preventive Medicine, The Alfred Centre, Monash University, Melbourne, VIC 3009, Australia; malcolm.sim@monash.edu.au

**Keywords:** occupational, injury, heat exposure, Australia, qualitative

## Abstract

Epidemiological evidence has shown an association between exposure to high temperatures and occupational injuries, an issue gaining importance with environmental change. The aim of this study was to better understand contributing risk factors and preventive actions based on personal experiences. Interviews were conducted with 21 workers from five Australian states using a critical phenomenological approach to capture the lived experiences of participants, whilst exploring contextual factors that surround these experiences. Two case studies are presented: a cerebrovascular injury and injuries among seasonal horticulture workers. Other accounts of heat-related injuries and heat stress are also presented. Risk factors were classified as individual, interpersonal and organizational. In terms of prevention, participants recommended greater awareness of heat risks and peer-support for co-workers. Adding value to current evidence, we have provided new insights into the etiology of the health consequences of workplace heat exposure with workers identifying a range of influencing factors, prevention measures and adaptation strategies. Underpinning the importance of these are future climate change scenarios, suggesting that extended hot seasons will lead to increasing numbers of workers at risk of heat-stress and associated occupational injuries.

## 1. Introduction

It is becoming widely recognized that occupational health and safety (OHS) and productivity can be compromised in hot conditions, with several studies reporting on the phenomenon in Canada [1,2,3], the United States of America [4,5], Europe [6,7], Asia [8,9] and Australia [10,11,12,13,14,15,16]. As global temperatures rise and extreme temperatures become more common, prevention measures in workplaces will be essential.

The causal pathway linking high temperatures and health and safety incidents in workers is underpinned by altered behavior and the failure of the body’s ability to balance body heat gained, both metabolically and environmentally, with heat lost via thermo-physiological mechanisms (i.e., convection, conduction, radiation and the evaporation of sweat) [17,18]. However, as well as the thermal environmental hazard, heat risks can be affected by workers’ physiological conditions, psychosocial environment and available adaptation options [19]. Heat-stressed workers can display characteristics and symptoms such as fatigue and loss of concentration, which, if ignored can lead to: classic heat-related illnesses ranging in severity from mild to potentially fatal, and/or occupational injuries such as wounds, lacerations, burns and falls, as indicated in a model outlined in a previous study [20].

The relevant literature in this area contains mostly quantitative research, characterizing the positive association between high-temperature environments and increased occupational incidents. Studies have involved the analysis of large datasets of occupational health outcomes data, providing valuable evidence on the epidemiology of workplace heat injuries and vulnerable workers [11,13,21,22]. Additionally, worker surveys and field studies have been conducted to elucidate the phenomenon of heat-related incidents in the workplace [19,23,24]. The use of data from multiple sources [25] may provide a broader perspective on how and why these injuries occur in hot conditions. However, few studies have explored the personal experiences of injured workers to add context to underlying influences and extend the evidence base for preventive measures.

The aim of this study was to investigate first-hand accounts of occupational heat-related injuries and incidents through interviews with affected workers. Using a critical phenomenological approach [26,27,28], we undertook a risk factor analysis based on workers’ personal experiences and discuss preventive actions based on participants’ responses. This knowledge will enhance the current understanding of OHS risks in high-temperature environments, and may inform interventions and evidence for policymakers in industry and government.

## 2. Materials and Methods

Using a qualitative study design, interviews were conducted with individuals with a history of working in hot environments. Recruitment of participants within South Australia and other Australian states involved emailing information about the research widely to government regulators, unions, organizations and industry groups for dissemination amongst their networks. The research team’s existing contacts in health and safety were used to aid in the distribution of information. Flyers were placed in local worksites and a website was developed, providing contact details for the research team. Interested persons who contacted the researchers were provided with further information about the aims of the project and confidentiality issues. For those willing to participate, a suitable time and venue for a face-to-face or telephone interview was arranged.

At the beginning of the interview, demographic information was obtained from the participants. Following the provision of signed informed consent, interviews were audio-recorded. The interview guide contained questions relating to the participant’s work and details of the heat-related incident(s) they wished to discuss. Participants were prompted to talk about the prevailing conditions beforehand and the consequences of the injury. Questions also covered organizational issues such as whether heat-related training was available, work practices, and allowable adaptation strategies. Finally, participants were asked about their suggestions that may help prevent similar injuries or incidents occurring at workplaces in hot conditions.

De-identified recordings were transcribed by two members of the research team (AH, SW) and read several times by the first author. Notes were made and sections were highlighted in the first part of the analysis. Transcripts were imported into NVivo version 11 software (QSR International Pty Ltd., Doncaster, Victoria, Australia) and coding of recurrent themes into ‘nodes’ was undertaken in an inductive, data-driven manner [29]. A conventional qualitative content analysis [30] was used to interrogate the data and capture contextual meanings. Three members of the research team (AH, SW, SH-E) used peer debriefing [30] to discuss the coding and the underlying data to refine and provide a more nuanced account of the themes.

An individual’s health behavior, and in this case, OHS behavior, can be influenced greatly by their surrounding environment. Indeed, people’s experiences at work are not sequestered from underlying economic and social conditions operating in and shaping their workplaces. For instance, we may reasonably assume that employees’ capacity to work safely is subject to management’s definition of what constitutes a safe or unsafe workplace, and these definitions are themselves rationalized by a broader range of historical cultural, political and industrial relations dimensions. Analysis of workers’ phenomenological accounts alongside socio-cultural contexts extends a traditional phenomenological focus on first-person experiences and brings to bear insights into how workers’ behaviors in the heat are bounded by sociocultural processes, including workplace norms and the application of economic rationalism. Resonating with the philosophical framework of critical realism [31,32], this approach treats workers’ accounts as interpretations of events, but can still garner important insights into the interpreted ‘reality’ of workplace contexts and processes, and how these pertain to workers’ ability to stay safe in the heat [33]. We therefore applied a critical phenomenological [26,34] lens to the data to help unpack levels of factors influencing injury risk [35]. Finally, themes were categorized into a coding frame stemming from the original research questions [29].

Ethics approval for the study was gained from The University of Adelaide (Ethics Approval No. H-2016-085), Queensland University of Technology, Monash University and the University of Western Australia.

## 3. Results

Twenty-two interviews (12 face-to-face and 10 by telephone) were conducted between March 2017 and February 2018. One interview was excluded from the study as the participant’s injury was unrelated to their occupation. Most were single interviews, one was with two participants and another was an informal pre-work discussion with a group of around 20 workers, although few contributed to the discussion. The study participants, from five Australian states, ranged in age from 23 to 68 years and were from industries including construction, transport, manufacturing, agriculture and emergency services (Table 1).

Using a descriptive phenomenological approach [28], themes were derived from the data in relation to: the participants’ experiences and consequences of heat-exposure, other contributing factors, and their suggestions for preventive measures.

### 3.1. Circumstances and Outcomes of Incidents

Participants spoke of heat exposure being associated with a range of incidents incurred by themselves or colleagues, and how incidents are prevented. Some recounted events that occurred at an earlier time when working in occupations different to their current role. “*All sorts of basic injuries”* were reportedly associated with working in hot conditions with hand injuries and trips commonly occurring. Other injuries mentioned included burns and blistered hands, lacerations and falls, one of which resulted in a compound fracture.

While in worst case scenarios injuries such as falls or lacerations may be life threatening in the extreme, even relatively minor injuries can have wide ranging effects. Physiologically and cognitively, one’s ability to function normally and safely can be affected due to changes in a worker’s behavior, work practice, or the wearing of personal protective equipment (PPE), and psychosocial effects can result if earning capacity is diminished. The following quotes allude to these issues:

*They have an incident such as a nick or a cut, when someone’s overheating they bleed like a pig …. because all the blood’s at the surface trying to cool you down*

(#17, manufacturing)

*It might just be a cut, it might just be falling over, but on the same note it can be fatal …. I can’t tell you how many times it has been just a cut or a fall or you know just a small injury but the effects are massive they really are*

(#1, construction)

Traffic accidents that occurred in the heat had life changing consequences for two participants. In one case, a worker cycling to work on a hot day was struck by a vehicle. Bleeding into the spinal cord occurred (described as being *“made worse by the heat”*) resulting in a spinal injury.

There were also reports of heat-related symptoms resulting in heat exhaustion, nausea, vomiting, headache, dizziness, loss of focus and concentration, light-headedness, sunburn, loss of consciousness or collapse, heat rashes, a brain injury (see Box 1) and severe heat cramps. The latter were described as:

*Uncontrollable cramping of the body and um, yeah, just lack of bodily function. It crippled me*

(#1, construction)

### 3.2. Contributing Factors

Unpacking the contextual factors for occupational heat-related incidents requires an acknowledgment of the complexities involving the direct and indirect influences on health and safety. As such, the themes were contextualized using a socioecological framework, with factors at the individual, interpersonal and organizational levels [35]. 

#### 3.2.1. At the Individual Level

At the individual or intrapersonal level, where factors affecting the self could be contributors to injury, the subthemes identified were: acclimatization, dehydration and fatigue, and age.

- Acclimatization.

When working in hot environments, people *“acclimatize to it after a week or so”*, according to a participant, whereas those experiencing the heat for the first time or after returning from leave can be more at risk. Notwithstanding, workers suffering heat-related symptoms who are unable to recover sufficiently prior to further heat exposure may *“get worse … as the time goes on”*. By contrast, a participant who was well acclimatized, having been employed as a rigger for many years without incident, was working near the top of a high tower on a very hot day when suddenly overcome with nausea and fatigue. His co-worker intervened to prevent the risk of falling.

- Dehydration and fatigue.

Dehydration was often mentioned as a contributing factor to heat stress symptoms including cramps and injury. In Box 1 the interviewee describes how thirst can be a poor indicator of dehydration. Fatigue was also said to be a precursor to heat-related incidents as it can lead to mistakes, poor judgement and an inability to concentrate, *“so all sorts of basic injuries start happening more often”*. It was claimed that there can be a heightened injury in the first few hours of the day during prolonged heat if workers start the shift fatigued following a hot, sleepless night.

- Age

Age can be a determining factor for injury risk as younger workers who reportedly often think “*they’re bullet proof*” generally have higher rates of injury, although older workers can also be at risk:

*The highest level of people who have any injuries, probably under 18s … the younger ones, because they’re just going at everything like a bull at a gate, but the next highest level would probably be the over 50s*

(#2, construction)

By contrast, a participant from the agricultural sector thought that the younger generation was more aware of farm safety “*than the old boys*”.

Box 1 **Participant’s account of heat-related brain injury.**

Len (not his real name) is a helicopter paramedic whose injury occurred two years previously. Len had been involved in stressful training and the retrieval of motor accident victims in hot weather in the lead up to the incident. By his own admission, he probably let himself get “*run down a fair bit*” during this time. On the day of the incident, he attended a vehicle accident requiring him to be in the sun for 3 to 4 h in temperatures close to 40 °C. He admits he did not drink enough during this time, yet never got thirsty. On the flight taking the patient to the trauma center, he became very unwell, with extreme dizziness and profuse vomiting. Upon landing, he recovered slightly with rehydration and a cool environment but had a persistent headache that worsened. The next morning, he began vomiting again. The headache persisted and Len was admitted to hospital 3 days later with suspected dehydration. With his blood tests being normal he was given further tests which showed he had incurred a cerebellar infarction. He spent 10 days in the coronary care/stroke unit with loss of balance and associated cardiac effects, after which he was sent to a rehabilitation unit where he spent six months recovering and learning to walk again. In Len’s words, dehydration and hyperthermia led to the heat stroke.

*On the day I was so dehydrated and so …hyperthermic that my blood was so thick that the little artery in that part of the cerebellum just blocked and the blood flow stopped causing the stroke.… Nobody could believe that such a drastic outcome could come from just dehydration.*

Fortunately, Len recovered completely and returned to work.

*I feel incredibly lucky. It could have killed me. If that … stroke had‘ve been in another part of the brain I could have been either completely incapacitated for life, or it could have killed me.*

When asked about recommendations for prevention of similar incidents. Len said:

*Your own wellbeing and fitness is … really important … the more unfit you are the more at risk you are I believe. … Make sure you look after yourself, you drink plenty of water, you keep well hydrated, you wear the appropriate clothing, always wear a hat, sunscreen. …you’ve got to make sure your organization is aware of what can happen and provides, um probably supervision. … Make sure that your colleagues are looking out for you as well and you’re looking out for your colleagues.*

#### 3.2.2. At the Interpersonal Level

Relations with others and peer pressure can influence cultural drivers of behavior towards health and safety. The theme identified at the interpersonal level related to concerns about self-presentation [36] in workplace situations, including the potential perception of oneself by others as being ‘weak’, or as lacking capacity to carry out the required work.

- Attitudes concerning self-presentation

While self-pacing and more frequent work breaks are well-established coping initiatives in hot conditions, participants said they can give an impression of weakness. Concerns were raised, for example, that taking a break may be viewed derogatorily, and it was recounted that terms such as “*sook*” or “*bludger*” had been previously directed at workers. In contrast, some exhibit stoicism and resist taking breaks, and a viewpoint mentioned was the need to change the Australian norms and culture around the attitude that “*you’ve got to man up to everything*”, which can be harmful to both physical and mental health. New workers in particular often feel they need to prove themselves and not take breaks:

*They feel under pressure, they feel like they have to keep delivering, they don’t stop, they don’t have the rests*

(#22, communication)

#### 3.2.3. At the Organizational Level

Workplace safety culture is very much dependent on the organization and its management, and in some cases, the physical location, as reflected in the following subthemes: workplace environment, workers’ lack of autonomy, workplace constraints, sub-contractors and seasonal workers.

- Workplace environment

Interviewees in this study were not only those who worked outdoors in high ambient temperatures, but also some whose experiences were in hot indoor environments. These included industries such as underground mining, transport and manufacturing, where a heat-generating machine was the main source of heat exposure. The tyranny of distance can be a concern if workers require medical attention when in isolated rural or remote regions of Australia. In these areas, temperatures can be extreme (e.g., *“48 degrees is not un-seasonally hot”*) and the closest medical center may be 6 to 7 h away. However, narratives from participants working in remote areas suggested that employers were well aware of the heat risks and all staff were trained senior first aiders *“with remote experience”.*

- Workers’ lack of autonomy

At the insistence of superiors, staff may need to continue to work at a normal pace despite dangerously hot conditions. Workers may therefore lack the ability to make autonomous decisions about their health and safety when such power inequality exists in the workplace (see Box 2), or when working with continually operating mechanical devices. In other instances, employees may feel indirect pressure to keep working without breaks within the parameters of implicit norms set by superiors. Young workers in particular can lack the confidence to speak up if they feel conditions are unsafe due to the power imbalance between workers and management:

*A young guy won’t speak up as much as an older, more experienced employee will. So they’ve got to be able to have the confidence to be able to communicate and speak up to their supervisor’s team leader or someone around them*

(# 19, retail)

Several participants mentioned the need for good supervision and an understanding by superiors that thermo-physiological limits can be reached in high-temperature environments, compromising OHS. It was said that good supervisors “*watch their blokes*”, ensuring their wellbeing, monitoring when they need a break and recognizing that work may take longer than normal in hot conditions:

*… but an understanding from a boss’s perspective, that we’re not, we’re not machines, that we’re humans and humans have limitations, and that ongoing effect of, for quality work to be done, sometimes leaving an hour earlier, and that understanding that it’s OK to leave an hour earlier, because tomorrow … you can still perform at a better level than if you push through for that one or two extra hours*

(#1, construction)

A lack of workers’ autonomy also exists around the mandatory wearing of personal protective equipment (PPE). Specifically designed to protect the wearer and reduce the risk of exposure to hazards, PPE can add to the body’s heat load if heat loss is impeded. For underground miners, the thick protective clothing, albeit made from natural fibers, reportedly led to “*horrendous skin rashes*”. For safety reasons, workers are generally unable to exercise discretion in the wearing of PPE despite the heat. However, a young scaffolder in this study thought he should be allowed to wear shorts in hot weather, while a traffic controller stated he was required to wear full coverage for the whole day with not even “*your cuffs rolled up”*, saying he would willingly sign a waiver about the threat of skin cancer if he could wear short sleeves or short pants. Protective eyewear can also be a problem, with a participant saying that safety goggles can fog up, and he questioned the need to wear them in certain instances:

*Safety goggles, they just fog up, and to me it just seems, it’s more unsafe to be wearing them*

(#3, construction)

Box 2 **Participant’s account of the effects of heat on seasonal migrant workers in Australia.**

Sally (not her real name) is a network coordinator with strong connections with the temporary migrant workforce in Australia. She has been alarmed at the high number of deaths and injuries in these workers in the agricultural industry and is concerned about potential links with heat stress, dehydration and hot, overcrowded, unsanitary accommodation with no fans or air conditioning. Some workers are “*grossly exploited*” with no ability to change employers. Although English proficiency and literacy skills can be low, inductions can be provided in English only. Work is often undertaken in hot conditions and Sally mentioned examples of deaths due to heat stroke. However, workers are not encouraged to stop for drinks and toilet breaks.

*There’s not enough breaks and there’s not enough access to water onsite and even, you know, stories of um having to buy the water from the farmer.*

*And if you’re on piece-rates and you down tools then you’re not going to make any money … Each of those factors influences how people behave in the heat. Um. Who’s controlling them, do they have any power to say no, um, what are the consequences if they do, and at the moment the consequences are, you know, well you’ll probably lose our job.*

Sally told of labor-hire contractors beating and threatening workers for “*not being fast enough*” but for these workers “*there’s not a culture of questioning authority*”. There is an economic imperative in the supply chain as “*there’s a lot of people making money*” out of the work being done, although the workers themselves take home little as they are charged high costs for transport and accommodation. Knife injuries are common but access to medical treatment means missing pay and a perceived sign of weakness. Furthermore, there is “*no right of stay*” in Australia and injured workers need to return home. This, said Sally, “*completely undermines the health and safety system*”.

Sally recommends a complaints mechanisms that does not lead to punishment, the adoption of accommodation standards for farm workers and an intermediary, “*non-government*” place, such as a migrant workers’ center, where workers can go to seek help and information on issues including health and safety.

- Workplace constraints

Economic constraints in a workplace can undermine OHS. This was reflected in the narratives with statements such as—*“it’s very, very hard to get this company that I work for to spend any money”, and “whenever there’s a downturn the first one to get the chop? Safety”.*

With adequate hydration being an important protective measure, it is concerning that for some workers, a lack of access to toilet facilitates (for example, when working in road construction) influences their hydration status. A participant said as a consequence he did not eat or drink when at work, and hence, “*all the time at work I’m dehydrated, constantly*”.

It was also stated that heat can elicit a huge financial cost to the industry if it is not addressed. A participant pointed out that organizations should consider the risks and cost effectiveness of work continuing in severe heat, as mistakes can be made by fatigued workers and injuries can result:

*It might just be a case of messing up a little piece of what we’re doing, which then has the flow on effect of costing the company money. Um, so it needs to be weighed up, you know, how cost effective is making people working in this heat. …………*

(#1, construction)

- Sub-contractors and seasonal workers

Participants indicated that in many industries, workers are now employed on a contract or sub-contract basis rather than in permanent positions. Here, there are economic drivers to keep working through hot conditions because, as pointed out by a participant in the construction industry, “*no work, no pay*”. Although taking a break from work is a recognized heat protective strategy, there can be an underlying economic incentive where contracted or self-employed workers receive payment only when a job is completed, in which case:

*They’ll just work right through, no matter what the conditions, do it as quick as they can.* ………………

(#6, transport)

Furthermore, contractors can be more at risk as they are often employed to do “*the more hazardous sorts of jobs*” and can have higher heat exposure. Sub-contractors and seasonal workers (see Box 2) may be employed through labor hire contractors and the level of safety training can be lower than for other workers, particularly if there are language barriers or a lack of understanding about safety cultures.

### 3.3. Prevention of Incidents

Like the causes and effects of workplace injuries, prevention is multifaceted. Below is a brief overview of the main subthemes emerging from the interview data. These focus on the need to educate workers about heat risk preventive measures, such as the need to look out for each other, take breaks and keep hydrated. The locus of responsibility is also discussed. Table 2 summarizes a number of effective strategies, together with recommendations from participants.

The need for heightened education about heat risks was a recurrent issue raised in the interviews. As workers can underestimate the effects unless personally experienced, the signs and symptoms of potentially fatal heat stress and associated injuries should form a substantial part of heat safety training:

*They don’t sort of really appreciate the dangers … they don’t really understand the ramification that if they do get heat stroke—it’s pretty bad*

(#5, manufacturing)

Safety training can vary in structure according to the work situation. Inductions are clearly necessary for all new workers including those contracted through labor-hire companies, with cultural consideration afforded to those with low literacy or whose first language is not English (see Box 2).

Daily ‘pre-starts’ or ‘toolbox’ meetings were often mentioned in the narratives, as shown in Table 2. These short talks at the start of the day can highlight the salient environmental hazards for the day ahead, thereby acting as a “*constant reiteration of workplace safety*” and *“continuous ongoing training and communication”*.

*Pre-starts starts off, OK are we wearing hats today, have we got our sunscreen on, you know if you feel, if these are the symptoms, we’ve got some signs made up, you know with urine color in it, … if it looks like that, it needs to look like this*

(#19, retail)

Clearly, the importance of adequate fluid intake both during and before working in the heat should be stressed. Participants’ accounts of the amount of water required varied according to the physical nature of the work and the prevailing environmental temperature. Notwithstanding, it was also mentioned that the consumption of too much water can lead to renal problems. Several participants mentioned the benefits of electrolyte supplements to replace those lost through sweating. However, there were also concerns that intake needed to be moderated as too much can cause physiological imbalances. Alternatively, consuming “*fruits with high water content because they’ve got natural electrolytes in them*” and having “*salty breakfasts*” were suggested to provide natural alternatives. Urine color and specific gravity tests can be used as indicators of hydration status. However, color charts can fade over time and can be a less reliable indicator as “*some vitamins and medications can color the urine*”.

Table 2 summarizes some of the key points that could be covered in heat stress training, with example quotes from participants. Included is a quote from a participant who, from his own experience, now recognizes the potential severity of the consequences and will not expect his staff to work beyond their limits in hot weather. Supervisors should be particularly aware of a worker’s need to self-regulate during long spells of hot weather, as shown in this quote which refers to cease-work temperature guidelines for industries such as construction:

*But for you to actually, to make it to the point where it turns 35 or 38, you still need to regulate yourself and you know, and take precautions to even get to those temperatures, because on a hot day not so much the first hot day, the second, the third the fourth, you’re already flat and struggling, when its 28*

(#1, construction)

Understanding the locus of responsibility for health and safety of workers helps to contextualize the notion of prevention. On the subject of training workers and supervisors, one of the interviewees said: “*you know yourself you’re responsible for your own safety*”. However, this becomes a dilemma when, as mentioned above, workers lack autonomy to undertake safe behavior. A health and safety officer stated that “*we all take ownership of safety… it’s not me, it’s all of us*”. Shared responsibility indeed supports the notion of colleagues looking out for each other and being aware of others’ potential change in behavior or mood that may be indicative of heat stress. Whereas responsibility can be personal, shared or organizational, this raises the question about blame if an incident occurs. On this topic a participant said:

*We also have to change this idea that as soon as there is an incident everybody has to overreact and start, basically I suppose, um putting it back on the injured party to actually, you know, like blaming them for the fact that there’s been an incident, or blaming the, you know, their company … So just recognize the fact that yes it is hot, … so we have to find a solution to it rather than trying to blame someone for it*

(#2, mining)

## 4. Discussion

This critical phenomenological study captured rich data about the lived experiences of occupational injuries and incidents incurred in hot environments, and contextualized these in reference to workplace processes, norms and conditions. Using a risk factor analysis, we have unpacked the details of heat-related incidents affecting over 20 workers in different Australian locations, and highlighted recommended preventive actions. Our findings highlight the complexities in causes and effects of heat-related incidents affecting not only outdoor workers, but also those working in non-cooled indoor environments, such as manufacturing and underground mining. This resonates with a previous study where it was noted that the majority of calls about heat stress to a regulator’s help line were from people concerned about conditions in hot indoor work environments [37].

Some of the participants in this study gave heartfelt accounts of their experiences, some of which they perceived as being life threatening or life changing. The types of incidents varied broadly and included injuries such as burns and lacerations, as reported in other studies [10]. Also reported were symptoms typical of ‘heat-related illnesses’, a term encompassing a spectrum of conditions [38]. That there is a blurred distinction between heat-related illness and injury is understandable given that heat exposure, dehydration and hyperthermia can be linked to both. As shown in this study and in the literature, heat-associated traumatic injury can also refer not only to external bodily injuries but also thermal damage to organs and tissues such as the brain, muscles [39] and kidneys [40].

To delineate the myriad factors that can contribute to heat-associated occupational incidents, we used a socioecological approach, recognizing that risk factors may be aligned with the individual, and/or be linked with interpersonal and organizational contexts [35]. Individual-level factors included the benefits of acclimatization that can render workers less likely to suffer from hyperthermia than those who are un-acclimatized [41]. Nevertheless, heat exposure effects can accumulate over several days, likely due to individuals working beyond their physiological levels of heat tolerance [42]. Dehydration can be a major contributor to muscle fatigue, core temperature elevation, declining physical performance and coordination and impaired cognitive function, thereby increasing injury risks. It is likely that dehydration may have been a precursor to many of the incidents reported by individuals in this study, as inadequate replacement of fluid lost through sweating can compromise the body’s ability to dissipate heat and maintain adequate blood flow to the muscles, skin and brain [18]. Interestingly, a recent study evaluating dehydration in occupational settings showed that most workers in sectors including construction, agriculture, manufacturing and tourism, usually started work in an evident state of dehydration [43]. This represents an additional source of risk for workers in hot environments, particularly if dehydration increases throughout the work shift.

At the interpersonal level, a key theme related to attitudes towards others who may be discerned as weak if faltering in the heat or taking breaks ‘unnecessarily’. Peer pressures and negative attitudes towards workers undertaking heat-protective behaviors have been reported in studies highlighting stereotypical attitudes that can prevail in workplaces [42,44]. Perceived ‘pressure’ to live up to a standard that equates taking breaks with ‘weakness’, may reflect a normative identity operating in workplaces that is at odds with safe work practices in the heat. Indeed, this finding resonates with the social psychological literature on impression management (for example, see references [36,45]), that proposes that people are intrinsically motivated to self-present in particular ways to impress others in different social contexts. In the current context, this can be associated with a worker’s preconceived need to ‘prove’ themselves and impress others by working on, despite the heat. This attitude is common in young workers who, as evidenced by the literature, are at higher injury risk in Australia’s heat [11,21,24].

Similarly, at the organizational level, there can be economic drivers to maintain productivity in hot conditions. With an imbalance of power between management and staff [44], workers can lack the autonomy to determine their safe work rate, rest breaks and access to water, thereby potentially increasing the risk of heat stress and injury. This was particularly salient in the narrative provided by a community worker who spoke of the poor working and living conditions endured by foreign seasonal workers in the horticulture industry. The participant knew of numerous injuries, and also heat-related deaths amongst these workers, an issue that has been raised in other forums [46,47]. The agriculture and construction sectors are typically characterized by a high number of migrant workers, with cultural aspects including linguistic and adaptation barriers that can contribute to the risk [23,48]. Furthermore, a recent study from Italy found that foreign workers in agriculture and construction had a different perception of heat than native workers, with less impact of heat on productivity, perhaps due to a poorer understanding of the related health risks [23]. Cultural and ethnicity issues can therefore represent important heat-related occupational vulnerability factors that at present have not been investigated in depth [48].

Personal as well as organizational economic imperatives can contribute to heat-associated health and safety risks for workers on low piece-work rates. A study by Underhill and Rimmer claims that “as long as piece rates fail to provide a living wage, workers will continue to take chances with their safety” [47] p. 41. Similarly, where stopping work affects remuneration, workers may be driven to continue working past their physiological limits in hot, hazardous conditions. This can be the case for workers on ‘non-standard work arrangements’ or hired through agencies for short-term contracts [49]. In today’s ‘gig-economy’ this is becoming more common as levels of permanent employment decrease in many industries. Challenges for safety managers can therefore arise with temporary and agency workers often having different perceptions of safety and higher injury rates than permanent employees [49].

Although a body of literature in Australia [10,50,51] and other countries [3,52] has identified heat as an injury hazard, heat policies generally focus on the prevention of heat-related occupational illnesses. These policies can overlook the fact that impaired mental function, alertness and motor control as a result of the body’s response to heat-stress [3] can lead to increased risk of injuries, the severity of which can be exacerbated by the heat, as reported by participants. Primary prevention measures which aim to prevent injury before they occur, include reducing exposure to the hazard (i.e., heat), and altering unsafe work behaviors that can increase the risk of injury [53]. Health promotion measures to limit heat exposure with rest breaks, and shaded or cooled areas for respite, ensuring access to cool drinking water, and acclimatizing workers to the heat [42], could therefore aid injury prevention. Additionally, it is advisable that workplaces be aware of upcoming heatwaves so that preventive measures can be actioned to minimize health risks to workers [16]. The Australian Bureau of Meteorology has on online heatwave service showing color-coded heatwave severity and heatwave forecast maps [54] that may be useful to workplaces [16]. A number of phone apps and an online assessment tool [55] are also available that enable workers to calculate and manage personal assessments of heat-stress risk.

Secondary prevention measures on the other hand, aim to detect and treat diseases/injuries promptly to reduce their impact [53]. In this study, we have identified the need for enhanced heat training to alert workers to the early signs of cognitive and physical signs of heat stress, and how to respond accordingly when an individual is affected. This knowledge may prevent progression of the condition to injury or serious heat illness, thereby acting as a secondary prevention measure. However, a recent heat exposure study in South Australia showed that only 43% of 749 surveyed workers and apprentices in selected outdoor industries had received heat-related training [24]. Training can take the form of inductions, regular sessions and/or ongoing discussions such as daily toolbox talks. The identification of the symptoms of heat illness and associated injury risks should be part of the training. Studies have shown the importance of ongoing reinforcement of health messages, and that workers support efforts to encourage others to follow safe work practices [56]. Nevertheless, in this study, it is clear that responses by colleagues can vary when workers take breaks to avoid heat stress. This is contrary to the notion that workmates should “*look out for each other*”. To address this issue, positive health messages should be incorporated in heat training, with both workers and supervisors informed that self-pacing in the heat as a prevention measure should be tolerated and viewed as ‘working smart’ [44] and cost effective, rather than a sign of weakness. The dangers of maladaptive workplace norms and stoic attitudes in the heat should also be discussed.

Finally, for individuals facing thermal stress, the risk of elevated core body temperature resulting from wearing PPE warrants attention. PPE provides primary protection from workplace hazards such as sun, heat, tools and chemicals, which can often pose added injury risks in hot conditions [10]. However, PPE made from impermeable fabrics that trap air between the skin and the fabric can be a secondary hazard if heat loss mechanisms are impaired, thereby challenging the body’s thermal homeostasis [57]. As a consequence, those required to wear full PPE in hot environments need to avoid under-hydration, overheating and fatigue. Studies have indicated that the optimization of work clothing (for example, ventilation cooling shirts [58] or garments incorporating a liquid coolant [59]) is a factor that may be of great importance for heat-stress mitigation in industrial settings where workers are exposed to elevated heat stress. Further research is required to investigate alternative fabrics suitable for PPE in hot environments and the feasibility of wearable cooling garments where possible.

Qualitative studies have limitations and small sample sizes invariably prompt critique about lack of generalizability. We argue that qualitative research is not intended to be generalizable yet adds valuable context to traditional epidemiological studies by helping to answer the ‘why’ questions. Where findings resonate with those in similar contexts, generalizability is increased [60]. A second limitation of this study is that some respondents experienced heat-induced symptoms not typically classified as injuries or illnesses. Nevertheless, accounts of these experiences were informative about heat exposure. Finally, some participants recounted incidents which occurred years previously, thereby raising the possibility of recall bias.

## 5. Conclusions

This critical phenomenological study has provided new insights into the context of workers’ injuries in the heat, and the adverse health and safety consequences of occupational heat exposure in Australia. Findings show that until experienced personally, there may be an under-appreciation amongst workers of the risks of severe and potentially life-threatening injuries and incidents that can occur due to heat exposure. Increasing the awareness amongst employers, supervisors, managers and workers that heat is an injury hazard, and of appropriate means of reducing risks with preventive and protective behaviors, will likely contribute to minimizing risks, particularly among vulnerable subgroups. While these measures focus heavily on health education, there is a clear intersect with health promotion. The safety culture of organizations should be such that there is support for employees’ autonomy to reduce their heat exposure and work pace as required to work safely in high-temperature environments. Although there is a triangulation of findings between this study and current epidemiological evidence, new information is presented about workplace constraints and interpersonal factors that can affect injury risks. Considering these aspects, there is a need to carry out increasingly customized prevention that takes into account the characteristics of each worker (for example, their metabolic rate, age, acclimatization level, medications used and personal heat exposure) as well as the type of work undertaken and the specific work environment [19,20]. This may inform heat awareness and OHS policies in workplaces facing longer and more intense hot spells.

## Figures and Tables

**Table 1:** Demographics of participants and interview details.

Gender	Age (Years)	Occupation/Industry ^1^	State ^2^	FTF/P ^3^	Incident(s) or Topic Discussed
M	39	Construction	SA	FTF	Heat cramps, heat exhaustion
M	59	Rigger	SA	FTF	Heat cramps, nausea, vomiting, fall resulting in broken arm
M	23	Scaffolder	SA	FTF	Non-specific
F	66	Consultant/Mining	WA	P	Heat stress, heat rashes
M	63	Manufacturing	Vic	P	Trips, disorientation, vomiting
M	42	Transport	SA	FTF	Dehydration, heat stress, collapse
F	45	Agriculture	SA	P	Fatigue
M, F	46, 57	Rigger, Inspector	SA	FTF	Dizziness, nausea, fatigue, collapse
M	61	Surveyor	SA	FTF	Headache, fatigue, nausea, vomiting, dizziness
M	55	Manufacturing	SA	FTF	Light headedness, tired
M	29	Council worker	SA	P	Thirst, tiredness, headache, mood change
M	54	Council worker	SA	P	Loss of consciousness
M	68	Transport	Vic	P	Motor vehicle accident, loss of consciousness
M	53	Transport	SA	FTF	Prevention measures
M	56	Manufacturing	SA	FTF	Dehydration, heat illnesses, fatigue, prevention measures
M × 2	25, 60	Transport	SA	FTF	Vomiting, fatigue, too much water
M	45	Retail	NSW	P	Loss of consciousness, dehydration
M	48	Education	WA	P	Spinal injury, traffic accident
F	52	Coordinator	NSW	P	Injuries, deaths in seasonal workers
F	42	Postal worker	Tas	P	Delirium, dizziness
M	43	Emergency Services	NSW	P	Vomiting, headache, brain injury

^1^ At time of incident; ^2^ SA—South Australia, WA—Western Australia, Vic—Victoria, NSW—New South Wales, Tas—Tasmania; ^3^ FTF—Face-to-face interview, P—Telephone interview.

**Table 2:** Participants’ examples and recommendations for the prevention of heat-related injuries.

Recommendation.	Example Quotes
Education of workers, supervisors, persons conducting a business or undertaking (PCBU)^1^	*Education is the main thing … and education of employers I think too, or PCBU* *Educate people, that’s the big one, because then that’s being proactive* *It was a real learning lesson and we actually toolboxed it.* *You’re not allowed into the field after I think September … August I think it is, without this sticker on your hat that says you’ve received heat induction … and it’s like 4 or 5 h process of induction into heat, so it goes pretty deep*
Looking out for each other	*We’ve been instilling enough knowledge in them all to keep an eye on each other um which um, probably has prevented any serious type heat-related injuries* *The supervisors … should be checking up on you, like making sure you’re alright and, you’re not getting too, like too hot or stressed or heat stroke, because … you don’t know it yourself when you start suffering from heat stroke, you don’t know it, it needs somebody else to, yeah come and check up on you*
Adequate hydration	*It’s not a reliable sign to depend on thirst to prompt you to drink* *Where its 40, 45 degrees in the shade, you would average up to 10 L a day, depending on what work you were doing*
Work-rest regimes	*A 20/10 split, so 20 min in the sun, 10 min rehydrating and catching your breath and just getting out of the heat*
PPE	*Make sure you’ve got your hat, and sunscreen, and long sleeves* *There’s no regulations around what these shirts are made of, or what those pants are made of. So you’ve got everything from cotton through polyester through, you know, all the different materials, some breathe better than others*
Better understanding of heat risks by employers	*Well the first thing would be for employers to understand, um, the working conditions, and if something takes a bit longer, well it takes a bit longer… it’s not having that pressure from your employers is, would be one of the first things, um, that I think of*
Devices and safety systems	Emergency Position Indicating Radio Beacon, which if activated *“they know where you are so they send someone straight out to you”* *We’ve got a cloud based safety management system. So all employees have access to that … in the workplace*
Heat policies	*Prior to this, my incident … didn’t have a formal heat mitigation policy and now they do as result*
First aid	*Having things like, you know, proper first aiders that understand heat stress is a good thing, and I think the first aid course, the basic first aid course doesn’t really cover that a great deal … heat stress could be its own first aid, you know even if it’s only half a day … it really should be its own component* *Everyone’s a senior first aider … with remote experience, so they know how to manage patients for a period of time while help arrives. So every single one of us in trained in that and that’s refreshed very 2 years*
Risk assessments	*Pre-starts, every like every morning you come in you have a 5 min, you have a quick talk … we actually go on to the (Bureau of Meteorology) website, … wind speeds … temperature’s going to be this, humidity is this, … stay out of the weather, try and stay inside, if you’re outside cover up, keep your fluids up* *We hadn’t had as much exposure with an incident like this where a guy actually fainted and an ambulance was required. So now we have risk assessments and we have a monthly toolbox each November or October on heat and heat stress*

^1^ PCBU = Person conducting business or undertaking.
